# Contact force sensing manual catheter versus remote magnetic navigation ablation of atrial fibrillation: a single-center comparison

**DOI:** 10.1007/s00380-023-02344-8

**Published:** 2024-01-08

**Authors:** Simon Schlögl, Klaudia Stella Schlögl, Philipp Bengel, Helge Haarmann, Leonard Bergau, Eva Rasenack, Gerd Hasenfuss, Markus Zabel

**Affiliations:** 1 Department of Cardiology and Pneumology, Heart Center, University Medical Center, Robert-Koch-Str. 40, 37075 Göttingen, Germany; 2 DZHK (German Center for Cardiovascular Research), Partner Site Göttingen, Göttingen, Germany

**Keywords:** Atrial fibrillation, Pulmonary vein ablation, Catheter ablation, Remote magnetic navigation, Contact force sensing

## Abstract

**Background:**

Data comparing remote magnetic catheter navigation (RMN) with manual catheter navigation in combination with contact force sensing (MCN-CF) ablation of atrial fibrillation (AF) is lacking. The primary aim of the present retrospective comparative study was to compare the outcome of RMN versus (vs.) MCN-CF ablation of AF with regards to AF recurrence. Secondary aim was to analyze periprocedural risk, ablation characteristics and repeat procedures.

**Methods:**

We retrospectively analyzed 452 patients undergoing a total of 605 ablations of AF: 180 patients were ablated using RMN, 272 using MCN-CF.

**Results:**

Except body mass index there was no significant difference between groups at baseline. After a mean 1.6 ± 1.6 years of follow-up and 1.3 ± 0.4 procedures, 81% of the patients in the MCN-CF group remained free of AF recurrence compared to 53% in the RMN group (*P* < 0.001). After analysis of 153 repeat ablations (83 MCN-RF vs. 70 RMN; *P* = 0.18), there was a significantly higher reconnection rate of pulmonary veins after RMN ablation (*P* < 0.001). In multivariable Cox-regression analysis, RMN ablation (*P* < 0.001) and left atrial diameter (*P* = 0.013) was an independent risk factor for AF recurrence. Procedure time, radiofrequency application time and total fluoroscopy time and fluoroscopy dose were higher in the RMN group without difference in total number of ablation points. Complication rates did not differ significantly between groups (*P* = 0.722).

**Conclusions:**

In our retrospective comparative study, the AF recurrence rate and pulmonary vein reconnection rate is significantly lower with more favorable procedural characteristics and similar complication rate utilizing MCN-CF compared to RMN.

## Introduction

Radiofrequency catheter ablation (RFCA) has emerged as the primary standard of care in patients with drug-refractory atrial fibrillation (AF) and has been proven more effective as antiarrhythmic agents in maintaining sinus rhythm on a mid- to long-term basis [1, 2]. The population of patients eligible for RFCA is steadily expanding, RFCA is applicable in patients with paroxysmal, persistent and long-persistent AF and its indication is guided primarily by the severity of patient’s symptoms [1, 2]. In our center, RFCA is a routine procedure for drug-refractory symptomatic AF since 2006. Later on, in accordance with the release of new guidelines, RFCA was also considered as first line therapy for patients with recurrent symptomatic AF in our center. Success rates of remote magnetic catheter navigation (RMN) ablation is still debated [3–17]. Most of the previous studies consisted of small patient numbers mainly with paroxysmal AF with a follow-up of less than 12 months. To this day, a direct multicenter prospective randomized trial is lacking, metanalyses [18–20] citing the previously mentioned studies. With advancing catheter technologies contact force sensing catheters were later available offering the chance of more effective lesion creation as cornerstone of effective pulmonary vein isolation (PVI) [2]. The two available direct comparison between RMN and manual catheter navigation (MCN) with contact force sensing (MCN-CF) showed controversial results by small patient numbers mainly with paroxysmal AF [11, 21]. So far, little is known about the efficacy of RMN ablation regarding AF recurrence compared to MCN-CF ablation. We hypothesized, that as proven in our previous retrospective analysis [22], impact of improved lesion creation with MCN-CF could lead to better clinical outcomes regarding AF recurrences. Our hypothesis was also encouraged by our previous data, directly comparing RMN to MCN without contact force sensing catheters [16]. The primary aim of the present retrospective study, therefore, was to compare the outcome of RMN versus MCN-CF ablation of AF with regards to AF recurrence in patients with either paroxysmal or persistent AF. Our secondary aim was to analyze periprocedural risk, ablation characteristics and repeat procedures.

## Methods

### Study design

**Fig. 1:**
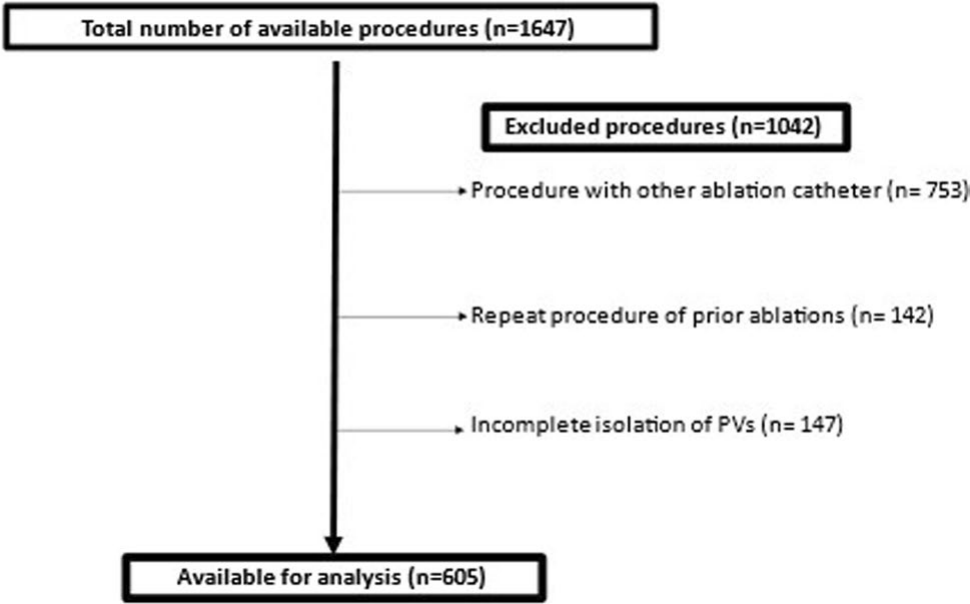
Screening of available cases. PV-pulmonary veins

We conducted a registry-based analysis of all consecutive patients with symptomatic drug-refractory AF undergoing percutaneous PVI in our center. The analyzed procedures (*n* = 1647) were performed between December 2008 and December 2018. Exclusion criteria comprised patients with previous AF ablation procedure before the index procedure in our center (*n* = 142), use of other ablation catheter than RMN or MCN-CF (*n* = 753) and incomplete isolation of pulmonary veins (*n* = 147) (Fig. 1).

### Patient’s characteristics

A total of 605 procedures (452 index procedures and 153 repeat ablations after the index procedure) by 452 patients with AF were included. 180 patients underwent RMN irrigated tip catheter ablation of AF (3.5 mm Navistar® Thermocool® RMT, Biosense-Webster, Diamond Bar, USA). We compared these patients with 272 patients with MCN-CF (Thermocool® SmartTouch® Surround Flow®, Diamond Bar, USA), respectively. Baseline characteristics of the two groups are shown in Table 1. In total, 66% of the patients presented with persistent AF at baseline, defined as AF lasting > 7 days or requiring cardioversion [1].

**Table 1:** Baseline characteristics of the patient groups

	MCN-CF (272)	RMN (180)	*P* value
Gender (male)	167 (61%)	110 (61%)	0.95
Age (years)	64.0 ± 10.8	63.4 ± 9.9	0.52
Paroxysmal AF	86 (31%)	66 (33%)	0.25
Persistent AF	186 (68%)	114 (63%)	0.25
BMI (kg/m^2^)	29.3 ± 5.1	27.8 ± 5.1	**0.03**
Hypertension	223 (82%)	141 (78%)	0.39
Coronary artery disease	60 (22%)	46 (25%)	0.42
COPD	22 (8%)	16 (9%)	0.73
Sleep Apnea	21 (8%)	20 (11%)	0.24
Hyperlipidemia	144 (52%)	81 (45%)	0.12
Diabetes	39 (14%)	27 (15%)	0.89
Left atrial size (mm)	45.6 ± 7.8	46.8 ± 6.5	0.06
LVEF (%)	52.5 ± 6.2	52.9 ± 5.0	0.44
Prior Cardioversion	194 (71%)	123 (68%)	0.59
Prior AADs	264 (97%)	170 (94%)	0.42

*AF* atrial fibrillation, *BMI* body mass index, *COPD* chronic obstructive pulmonary disease, *LVEF* left ventricular ejection fraction, *AAD* antiarrhythmic drugs

### Ablation procedure

All patients gave informed consent prior to the ablation procedure. The choice to RMN or MCN-CF RFCA was decided according to patient preference. In all subjects, left atrial (LA) thrombi were excluded by transesophageal echocardiography, and LA anatomy was acquired by contrast-enhanced high-resolution thoracic computer tomography prior to the procedure. All ablation procedures were performed with conscious sedation using intravenous sufentanil, midazolam and/or propofol under continuous monitoring of blood pressure and oxygen saturation. For the electrophysiological procedure, all catheters were advanced via the femoral veins. A 6F steerable decapolar catheter (Bard Dynamic Tip®, Bard Inc., Lowell, MA, USA) was positioned in the coronary sinus. After a fluoroscopically guided double transseptal puncture an SL1® sheath (St. Jude Medical, Inc., St. Paul, MN, USA) in the RMN group or an Agilis® deflectable sheath (St Jude Medical Inc., St. Paul, MN, USA) in the MCN-CF group were advanced into the LA. In the RMN group, a 3.5 mm open-irrigated, magnetic mapping and ablation catheter (Navistar® Thermocool® RMT, Biosense-Webster, Diamond Bar, USA) was advanced through the sheath into the LA, whereas in the MCN-CF group, a manually guided, open irrigated tip and contact-force sensing mapping and ablation catheter (Thermocool® SmartTouch® Surround Flow®, Biosense-Webster, Diamond Bar, USA) was used. After January 2010, a circular mapping catheter was initiated as a standard tool during every pulmonary vein ablation. The circular mapping catheter (Lasso®, Biosense Webster, Diamond Bar, CA, USA) was positioned within the PV ostium to monitor electrical activity during ablation and to verify electrical pulmonary vein isolation. Intravenous heparin was administered immediately after the transseptal puncture to maintain an activated clotting time (ACT) of 300–350 s throughout the procedure. Patients presenting with persistent atrial fibrillation underwent electrical cardioversion prior to mapping and ablation. Circumferential pulmonary vein ablation was performed using a three-dimensional mapping system (Carto® XP or Carto® 3, Biosense Webster, Diamond Bar, CA, USA) in conjunction with the integrated CT image of the LA and real-time fluoroscopy. To assure an accurate 3D model acquisition, respiratory gating was performed. In the RMN group, the Niobe II® magnetic navigation system (Stereotaxis) and a joystick-controlled motor drive (Cardiodrive®, Stereotaxis) were utilized for remote magnetic navigation of the ablation catheter, whereas in the MCN-CF group, the ablation catheter was guided manually. In the case of persistent AF, additional ablation lines were considered during the repeat ablation in case of persistent isolation of the pulmonary veins. The RMN system has been described in detail before [3]. Briefly, two permanent magnets located on either side of the procedure desk generate a magnetic field (0.08 Tesla) within the patient. The magnetic ablation catheter incorporates four magnets in the distal portion of the catheter. A change of the desired vector for catheter orientation on a computer screen results in alteration of the magnetic field generated by the permanent magnets and thereby corresponding deflection of the magnetic catheter within the heart. The joystick-controlled motor drive allows catheter advancement and retraction. Thus, the system provides complete remote catheter navigation for mapping and ablation. RF current was delivered for 30–60 s per lesion, applying 40 W (irrigation flow rate 30 ml/min) or 30 W at the posterior LA wall (irrigation flow rate 17 ml/min) with the generator (Stockert®, Biosense Webster) in a power-controlled mode and with an upper temperature limit of 45 °C in RMN group. An interlesion distance of ≤ 6 mm was aimed for. In the MCN-CF group, contact force was continuously monitored. According to manufacturer’s protocol, a contact force of 5–20 g was targeted during ablation. A force time integral (FTI) with an aim of 330 g seconds was used to determine acceptable lesions. Excessive tissue contact force (> 50 g) was visually indicated for safety considerations. Endpoint of the ablation procedure was the electrical isolation of all PVs defined as bidirectional conduction block. This was verified by the lasso catheter and a careful and repeated mapping for residual potentials around the entire circumference of the PV ostia, and pacing from multiple sites within the circumferential line. All pulmonary veins were examined at the end of the procedure resulting in waiting periods of > 30 min for the left superior pulmonary vein (LSPV) and left inferior pulmonary vein (LIPV) and ca. 5 min for right superior pulmonary vein (RSPV) and right inferior pulmonary vein (RIPV). No drugs were used to illicit triggers or uncover dormant isolation. All procedures were performed by the same experienced operators.

### Follow-up

After hospital discharge, patients were followed in our outpatient clinic and a 4-day continuous Holter electrocardiogram was repeated after 3, 6 and 12 months and on a 12-month basis thereafter. At each visit, subjects were asked for symptoms, documented arrhythmia recurrences, and current medication was assessed; 122 (27%) of the patients had an implanted cardiac device, which was interrogated in every visit. Furthermore, all patients were advised to present themselves immediately in case of symptoms suggestive for arrhythmia recurrence and obtain ECG documentation. An electrical cardioversion was performed prior to discharge in case of detected AF/atrial tachycardia (AT) recurrence post-interventional for AF episodes lasting longer than 6 h. Furthermore, in some cases antiarrhythmic drugs (AADs) (flecainide, propafenone, dronedarone, amiodarone) were continued for the next 3 months (blanking period) with termination of the antiarrhythmic medication after the blanking period. A documented AF/AT episode lasting longer than 30 s after the blanking period was considered a recurrence. Additional diagnostic information (e.g., echocardiogram, chest X-ray/computer-tomography) was acquired if symptoms were suggestive of procedure-related complications (e.g. pericardial effusion, pulmonary vein stenosis, phrenic palsy).

### Statistical analysis

Variables are expressed as mean ± standard deviation (SD) if normally distributed, or as percentage or median value with 25th and 75th percentiles interquartile range (IQR). Differences in the frequency of characteristics were assessed by independent samples Student’s t-test for continuous variables. Chi-square statistic (or Fisher’s exact test if applicable) was used for discrete/categorical variables. Probability of AF recurrence was based on the time to first AF recurrence after the index procedure determined by Kaplan–Meier analysis with Mantel-Cox (Log-Rank) test. Time to first AF recurrence was plotted as a Kaplan–Meier curve. If a crossover between RMN and MCN-CF groups occurred, follow-up was censored. A Cox proportional hazards model with multiple variables was performed to identify predictors of AF recurrence in a multivariable analysis at follow-up. All tests were performed with a two-tailed significance level of 0.05. We used SPSS 23.0 (SPSS, Inc.) for data analysis.

## Results

### Baseline, procedure

Acute success rate of RMN-based approach proved to be lower compared to MCN in our previous publication [16]. In our total retrospective cohort, acute success proved to be lower in the RMN cohort (74%) compared to MCN-CF cases (99%) (*P* < 0.001). However, as mentioned in the study design section, incomplete PVs were excluded from further analysis. At baseline, there were no significant differences between the groups except body mass index (Table 1). The ablation characteristics and follow-up data of both groups are summarized in Table 2. In the RMN group, total procedure time and radiofrequency application time were significantly higher despite same number ablation points compared to MCN-CF group. The total fluoroscopy time and fluoroscopy dose were significantly higher for RMN compared to MCN-CF.

**Table 2:** Ablation characteristics and follow-up data

	MCN-CF (272)	RMN (180)	*P* value
Total procedure time (min)	113.8 ± 29.2	216.7 ± 52.5	** < 0.001**
Fluoroscopy time (min)	14.7 ± 7.6	16.9 ± 11.9	**0.016**
Fluoroscopy dose (Gycm^2^)	2035 ± 4770	5270 ± 3954	** < 0.001**
Radiofrequency time (min)	23.7 ± 7.4	55.52 ± 15.9	** < 0.001**
Number of ablation points	57.2 ± 20.8	59.7 ± 21.5	0.20
Complete isolation of all PVs (%)	272 (100%)	180 (100%)	1.00
Lost to follow-up	6 (2.2%)	0 (0%)	0.08
Mean follow up (months)	18.7 ± 16.8	18.8 ± 23.3	0.28
Number of procedures	1.2 ± 0.4	1.3 ± 0.4	0.20

*PV* pulmonary vein

### Safety

A major peri- or post-procedural complication presented in total of 13 cases (2.1%): 6 (2.4%) in the RMN group and 7 (1.9%) in the MCN-CF group experienced major peri- or post-procedural complications without statistical difference between the groups. (*P* = 0.722) (Table 3).

**Table 3:** Safety endpoints

	MCN-CF (355)	RMN (250)	*P* value
Postprocedural effusion after ablation	1 (0.2%)	2 (0.8%)	n.s
Post-procedural stroke	0	0	n.s
Phrenic nerve palsy	2 (0.5%)	0	n.s
Postprocedural groin complication	1 (0.2%)	4 (1.6%)	n.s
Atrio-esophageal fistula	0	0	n.s
Pericardiocentesis after failed TsP	3 (0.8%)	0	n.s
Total	7 (1.9%)	6 (2.4%)	n.s

*TsP* transseptal puncture

### Efficacy

**Fig. 2:**
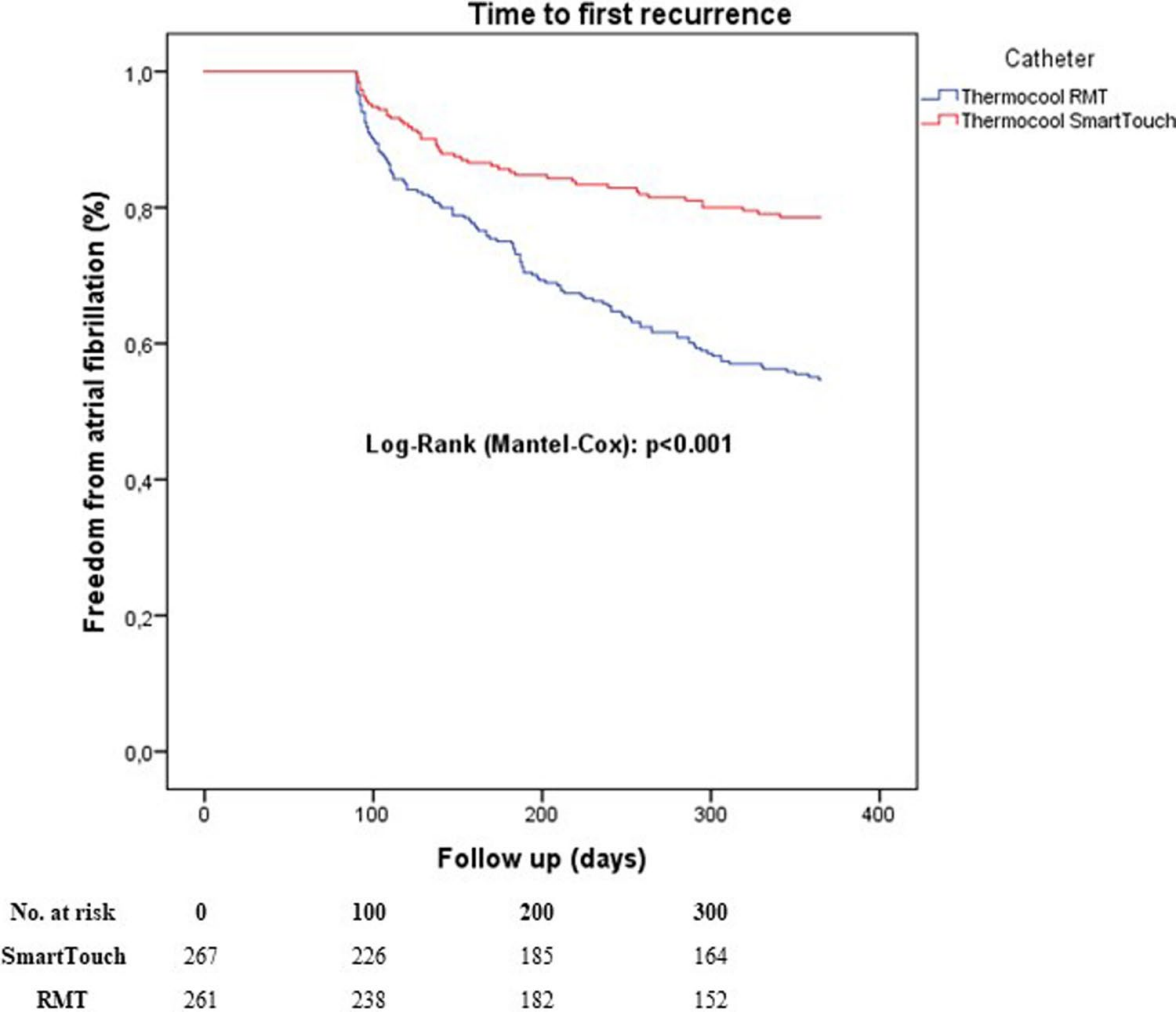
Time to first recurrence after last ablation including incomplete PVI cases

Before exclusion of cases with incomplete PVI, freedom from AF/AT was significantly lower after RMN compared to MCN-CF (*P* < 0.001, Fig. 2).

**Fig. 3:**
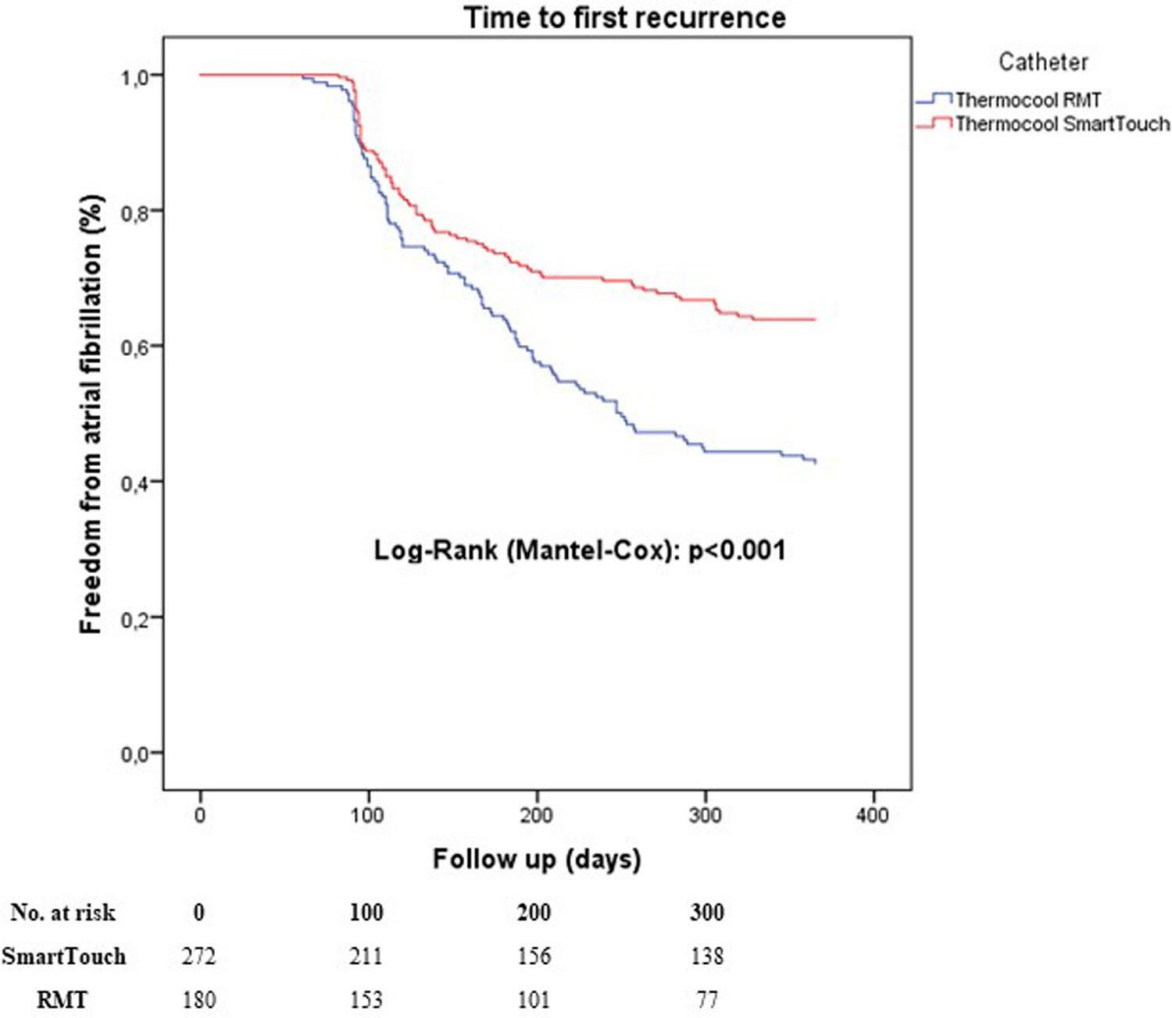
Time to first recurrence after first ablation excluding incomplete PVI cases

**Fig. 4:**
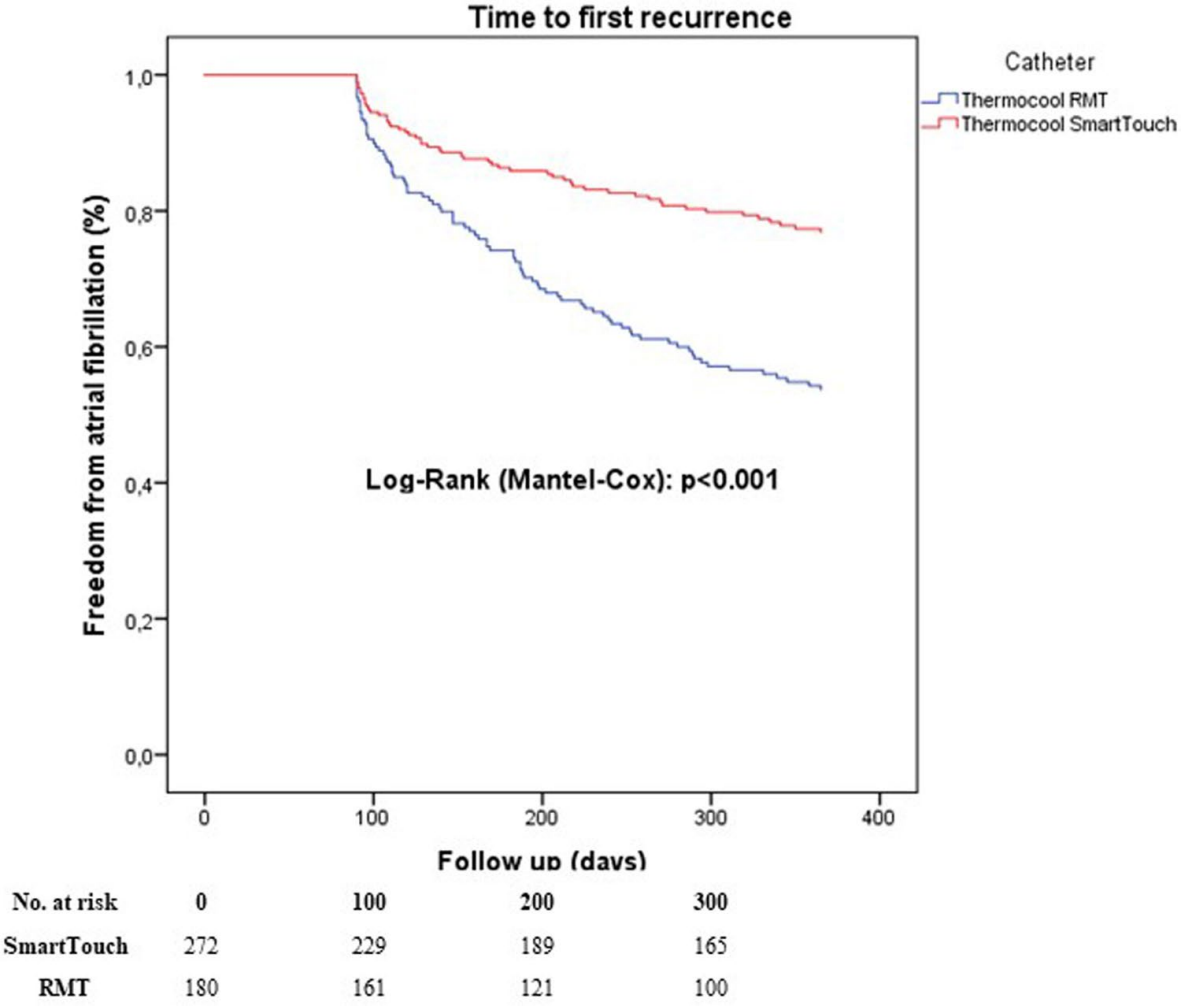
Time to first recurrence after last ablation excluding incomplete PVI cases

**Fig. 5:**
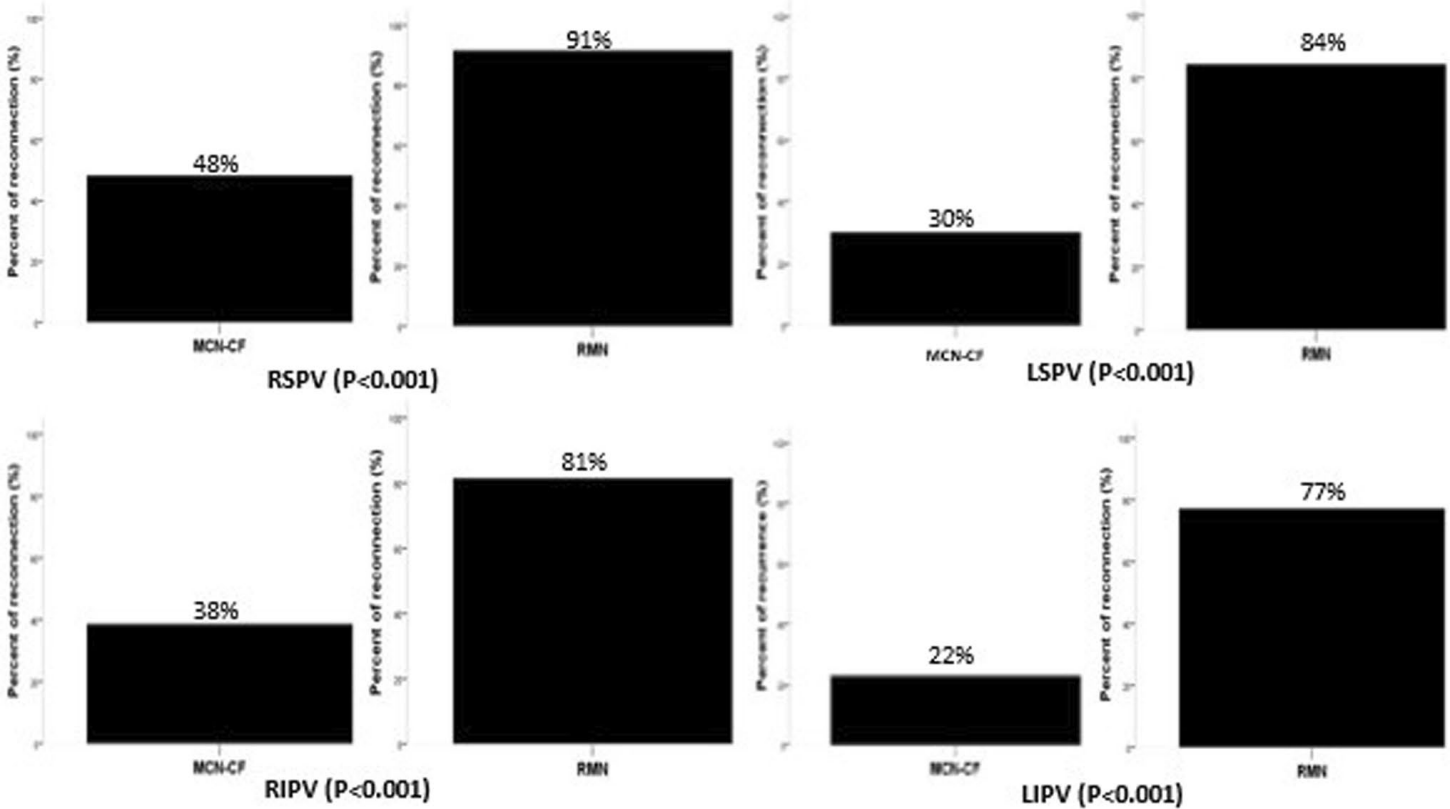
Percent of pulmonary vein reconnection by the redo procedures. LSPV left superior pulmonary vein, LIPV left inferior pulmonary vein, RSPV right superior pulmonary vein, RIPV right inferior pulmonary vein

After exclusion of incomplete PVI cases, time to first recurrence after the first ablation procedure was significantly shorter in the RMN group compared to MCN-CF group (*P* < 0.001, Fig. 3). This difference was not changed after considering redo procedures *P* < 0.001, Fig. 4). A multivariable Cox regression analysis was calculated to define predictors of AF recurrence after the procedure. Remote magnetic catheter navigation and left atrial diameter were associated independently with a higher risk of recurrence of AF (Table 4). The analysis of 153 repeat ablations showed significantly higher rate of reconnections after RMN ablation (*P* < 0.001, Fig. 5).

**Table 4:** Proportional hazard analysis for primary endpoint

Parameter	Univariate analysis			Multivariate analysis		
	HR	*P* value	95% CI	HR	*P* value	95% CI
RMN Catheter	3.83	** < 0.001**	2.44–5.99	1.651	** < 0.001**	1.294–2.106
LA diameter	1.05	** < 0.001**	1.02–1.08	1.022	**0.013**	1.005–1.039
LVEF	0.96	0.08	0.92–1.00	0.992	0.390	0.973–1.011
Age	1.01	0.09	0.99–1.03			
COPD	1.75	0.16	0.80–3.81			
Hypertension	1.30	0.22	0.83–2.18			
CAD	1.33	0.23	0.83–2.13			
Cardioversion	1.26	0.27	0.83–1.92			
Persistent AF	1.22	0.32	0.81–1.84			
BMI	0.98	0.40	0.94–1.02			
Sleep Apnoea	1.33	0.41	0.66–2.70			
Diabetes	0.80	0.42	0.46–1.37			
Female Sex	1.14	0.51	0.76–1.70			
Hyperlipidaemia	1.06	0.76	0.71–1.56			
Smoking	0.99	0.99	0.61–1.60			

*AF* atrial fibrillation, *CF* contact force, *LA* left atrial, *COPD* chronic obstructive pulmonary disease, *BMI* body mass index, *CAD* coronary artery disease, *LVEF* left ventricular ejection fraction

## Discussion

### Main finding

The main finding of this retrospective registry-based study is a significantly higher one-year AF recurrence rate of RMN guided PVI after the last procedure compared to the MCN-CF guided approach in a real-life cohort of AF patients. To the best of our knowledge, our study is the largest cohort of patients with the most ablation cases comparing RMN guided approach to MCN-CF; showing a significant difference in one year success after adjusting for multiple confounders of PVI success rates. Moreover, our findings are also supported with the analysis of pulmonary vein reconnections by the redo procedures. Importantly, compared to previous studies which included a majority of patients with paroxysmal AF, most of our patients presented with persistent AF.

### Literature overview

Feasibility of RMN-guided RFCA in AF has been well demonstrated in earlier studies [3, 4]. Comparisons of RMN-guided PVA with conventional manual catheters without contact force sensing were often conducted in rather small patient groups with limited follow up time and with a majority of patients presenting with paroxysmal AF [3–10, 12–15]. Recent meta-analyses included all available studies to date [18–20]. Based on the available data, Proietti et al. questioned if previous studies would be conclusive on the effectiveness of RMN-PVI [20]. Our previous data showed better efficacy of manual navigation without contact force sensing compared to RMN [16]. Based on previous metaanalyses [23–25] and our retrospective data [22], we hypothesized that manual catheters with CF may yield a better clinical efficacy compared to manual catheters without CF. In the literature, we were able to identify two studies comparing at least 50 patients with MCN-CF to RMN. Weiss et al. compared 312 patients with MCN to 315 patients with RMN [11]. However, only 59 patients were treated with MCN-CF. MCN-CF subgroup showed significantly more AF recurrence compared to RMN group after 12 months of follow-up inferior clinical efficacy (*P* = 0.02). However, a detailed clinical characteristic of MCN-CF subgroup compared to RMN group was lacking and recurrence factors were not analyzed on a multivariate basis. Jez et al. compared 57 patients with RMN to 89 patients with MCN-CF [21]. This study showed no difference between AF freedom between RMN and MCN-CF (paroxysmal AF: 60.8% RMN and 73% in MCN-CF group, *P* = 0.42; persistent AF: 69.6% RMN and 75% MCN-CF (*P* = 0.77)), however, MCN-CF group showed slightly better freedom from AF. However, total follow-up was only 6 months.

### Efficacy endpoint

Acute success rates after RMN ablation showed significant variation between previous RMN publications. Nine studies did not report any data about acute success rates (e.g. complete isolation at the end of the procedure). Five publications reported only cases with complete isolation of all pulmonary veins between groups. Lastly, nine publications reported an exact rate of acute success after RMN ranging between 43 and 99% [19]. In our previous study, RMN proved to be inferior in case of acute success compared to MCN-CF [16]. In our current retrospective data cohort, acute success proved to be lower after RMN (74%) compared to MCN-CF (99%), leading to higher AF recurrence after the last procedure (Fig. 2). To rule out a potential bias through lower acute success, cases with incomplete PVI were excluded from the ultimate analysis. However, this did not change the results showing significantly higher AF/AT recurrence after RMN cases.

In the context of possible explanations for the lower clinical efficacy of RMN-PVI, two main factors should be mentioned. Firstly, the cornerstone of successful PVI is the electrical isolation of the pulmonary veins. In cases, where this goal is not achieved, PVs are either partly or remain only temporarily isolated, leading to AF recurrence. As it was proven by previous studies [17, 26], flexibility of the magnetic catheter shaft may result in a reduction of the maximal force applied to the tissue. During the RMN procedure, circular mapping catheter-guided pulmonary vein isolation can be demanding in some cases, especially on the subject of the right pulmonary veins, due to anatomical complexities and the relationship of the ostium of the right inferior PV to the insertion of the transseptal sheath to the LA, thus resulting in longer procedure time and occasional unfeasible isolation of the right pulmonary veins [10]. In accordance with these previous findings, analysis of redo procedures showed more recurrences after RMN compared to MCN-CF. Moreover, recurrence rate in the RMN group was more pronounced by the right pulmonary veins, especially by the right superior pulmonary vein (Fig. 5).

Secondly, radiofrequency application time was significantly higher by the same rate of ablation points in the RMN group, probably reflecting lower tissue contact force. The efficacy of RMN ablation in creating ablation lesions has been discussed before [27]. In line with these findings, Béssiere et al. reported that the contact force averages approximately 6 g, with similar results obtained using simulated transvenous and retrograde approaches and with 0.08 and 0.10 T magnetic field [28], thus obtaining lower values than during standard manual ablation procedures (e.g. 10–12 g) [29]. Translating into the clinical efficacy, success of PVA at 12-month FU was significantly higher in patients with an average contact of 20 g compared to an average contact force of 10 g [30, 31]. We could presume that RMN was lacking efficacy to create a durable unexcitable ablation line due to reduced maximal force applied, making patients more prone to recurrence.

Lastly, according to the literature overview and our previous publication, acute success, defined as the complete isolation of the pulmonary veins is between 43 and 99% after RMN guided ablations [16, 19], suggesting an acute efficacy issue of RMN by pulmonary vein isolation. Possible explanations of the limited acute success by RMN were discussed in the previous two points. Interestingly, this issue is not known by ventricular ablation leaving us to speculate about the concrete mechanism and giving ground for further studies.

In our study, use of RMN was associated with longer fluoroscopy time, higher fluoroscopy dose and longer ablation procedure time. To rule out a potential learning effect by the RMN group, we compared the first 50% of the ablations to the second 50% of the ablations. There was no significant difference regarding total procedure time (211 ± 47 min. vs. 222 ± 57 min.; *P* = 0.141) and total ablation time (55.45 ± 15.8 min. vs. 55.5 ± 16.2 min; *P* = 0.957) between the first 50% and last 50% of ablations. In comparison to previous publications, our findings show improved procedural characteristics with MCN-CF catheters [16, 25], thus indicating that the latest generation of catheters with contact force sensing technology outperform RMN in the aspect of fluoroscopy and procedural characteristics. Our data also indicate that more radiofrequency current has to be delivered when utilizing RMN-guided ablation as compared to the MCN-CF approach to achieve ablation lesions, in line with the probable explanation of lower possible contact force during RMN ablation discussed in the previous points.

### Safety

The complication rates in our study data are comparable to those in worldwide surveys and previous metanalysis [25, 32]. Procedural safety was an undisputed advantage of magnetic catheter navigation in comparison to manual catheter navigation without contact force sensing: RMN was associated with almost 50% lower risk of major procedural complications compared with MCN without CF [25]. In our data, major complication rate failed to reach statistical significance between groups, showing safety improvements with the utilization of CF-sensing technology. Lower applied contact force during RMN is considered the major factor for reducing shear atrial wall stress and deformation, preventing pericardial effusion and tamponade. However, according to our data, with the application of contact force monitoring this safety advantage over MCN catheters disappears.

### Limitations

Firstly, we conducted a registry based, single-center retrospective study. AF recurrence rates were to some extent dependent on the patient’s and general practitioner’s awareness and responsiveness. Thus, asymptomatic episodes of AF may have been missed. Secondly, patients analyzed in this study were not completely ablated in the similar time span: RMN cases were mainly ablated between 2008 and 2014 and MCN-CF cases mainly between 2014 and 2018. Furthermore, the same experienced operators performed all of our procedures. However, as procedural experience increases, operators develop more skill and experience which may lead to improved outcomes. We cannot fully exclude that this aspect also plays a role. Our study did not incorporate ablation index or Carto Visitag® module with stability settings as these technical improvements were not readily available in our center at the time of this study. Lastly, our study does not include CF sensing by RMN catheters as they were not available in our center by the time of the study.

## Conclusions

In this registry-based retrospective comparison, pulmonary vein ablation using manual catheter navigation with contact force sensing has a lower rate of pulmonary vein reconnections and AF recurrence rates and decreased procedural time, ablation time, fluoroscopy time and fluoroscopy dose with the same safety features as compared to remote magnetic navigation.

## Data Availability

Data available on request due to privacy/ethical restrictions.

## Abbreviations

RMN: Remote magnetic catheter navigation
MCN-CF: Manual catheter navigation in combination with contact force sensing
AF: Atrial fibrillation
vs.: Versus
RFCA: Radiofrequency catheter ablation
PVI: Pulmonary vein isolation
LA: Left atrial
ACT: Activated clotting time
FTI: A force time integral
LSPV: Left superior pulmonary vein
LIPV: Left inferior pulmonary vein
RSPV: Right superior pulmonary vein
RIPV: Right inferior pulmonary vein
AT: Atrial tachycardia
AADs: Antiarrhythmic drugs
SD: Standard deviation
IQR: Interquartile range
